# Prevalence, Diagnosis, and Treatment of Cardiac Tumors: A Narrative Review

**DOI:** 10.3390/jcm14103392

**Published:** 2025-05-13

**Authors:** Mohamed Rahouma, Hosny Mohsen, Mahmoud Morsi, Sherif Khairallah, Lilian Azab, Maya Abdelhemid, Akshay Kumar, Magdy M. El-Sayed Ahmed

**Affiliations:** 1 Cardiothoracic Surgery Department, Weill Cornell Medicine, New York Presbyterian Hospital, New York, NY 10065, USA; dr.adly90@gmail.com (M.M.); smk4005@med.cornell.edu (S.K.); lilianazab25@gmail.com (L.A.); maya.abdelhemid@stonybrook.edu (M.A.); 2 Surgical Oncology Department, National Cancer Institute, Cairo University, Cairo 11796, Egypt; 3 Department of Cardiothoracic Surgery, Faculty of Medicine, Beni Suef University, Beni Suef 62511, Egypt; hosny.abdelhalim@gmail.com; 4 Department of Biology and Psychology, Stony Brook University, Stony Brook, NY 11794, USA; 5 Cardiothoracic Surgery Department, NYU Langone Health, New York, NY 10016, USA; akshay.kumar2@nyulangone.org; 6 Cardiothoracic Surgery Department, Mayo Clinic, Jacksonville, FL 32224, USA; ahmed.magdy@mayo.edu; 7 Surgery Department, Faculty of Medicine, Zagazig University, Zagazig 44519, Egypt

**Keywords:** cardiac tumors, multimodal imaging, surgical resection, autotransplantation, oligometastatic disease

## Abstract

Cardiac tumors, though rare, present significant diagnostic and therapeutic challenges due to their heterogeneous nature and anatomical complexity. This narrative review synthesizes current evidence on prevalence, diagnostic modalities, and management strategies for primary and metastatic cardiac tumors. Echocardiography, cardiac MRI, and CT remain cornerstone imaging tools for differentiating tumors from non-neoplastic masses, while advances in PET/CT and tissue characterization techniques refine staging and treatment planning. Surgical resection with clear margins (R0) is critical for resectable tumors, particularly benign myxomas, though malignant tumors like sarcomas require multimodal approaches combining surgery, radiotherapy, and systemic therapies. Emerging strategies such as heart autotransplantation and staged resections offer promise for complex cases, while oligometastatic disease management highlights the role of stereotactic radiotherapy and immunotherapy. Key challenges include standardizing resection margins, optimizing neoadjuvant therapies, and addressing high recurrence rates in malignancies. Future directions emphasize integrating AI-driven imaging analysis, molecular biomarkers, and genomic profiling to personalize therapies, alongside global registries to enhance data on rare tumors. Equitable access to advanced diagnostics and multidisciplinary collaboration are essential to improve outcomes. This review underscores the need for standardized guidelines, technological innovation, and patient-centered research to address gaps in cardiac oncology.

## 1. Introduction

Cardiac tumors are rare, with an incidence of approximately 0.03%, making the available studies about their management relatively limited, particularly for malignant tumors [1]. Cardiac masses encompass a broad spectrum of lesions, including neoplastic masses (primary tumors, both benign and malignant, and metastatic tumors) and non-neoplastic masses (such as thrombi, vegetations, hamartomas, and calcified amorphous tumors) [2]. According to the 2021 WHO classification of cardiac tumors, cardiac masses should be carefully distinguished into pseudotumors (non-neoplastic) and true neoplasms (benign or malignant) [3].

Among primary cardiac tumors, 75% are benign and 25% are malignant [1,4]. Atrial myxoma remains the most common primary cardiac tumor in adults, whereas rhabdomyosarcoma is the most frequent in pediatric populations [1,2]. It is important to emphasize that cardiac metastases are significantly more prevalent than primary cardiac tumors, occurring in up to 10% of patients with advanced cancers [3]. The most common cancer to metastasize to the heart is lung (37%), hematological malignancies (20%), breast (7%), and esophageal cancer (6%) [2].

**Figure 1:**
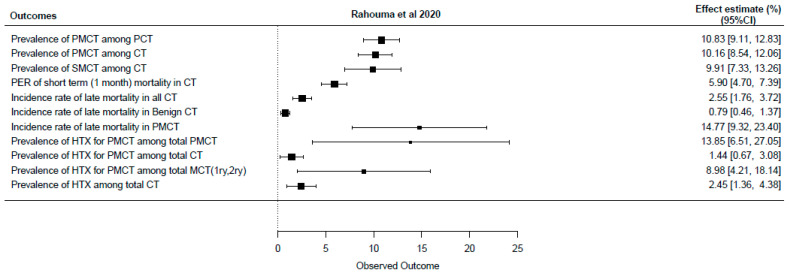
Forest plot of most recently published meta-analysis (CT: cardiac tumor, HTX: heart transplantation, PER: pooled event rate, PMCT: primary malignant cardiac tumor, SMCT: secondary malignant cardiac tumor) [5].

Despite the rarity of cardiac tumors, several meta-analyses have attempted to synthesize available evidence on their prevalence and outcomes. A recent comprehensive meta-analysis including data from 74 studies and 8849 patients provided updated insights into tumor types and mortality trends [5]. Earlier meta-analyses focused on narrower topics, such as coronary disease in myxoma patients [6] and pediatric tumor mortality [7], but showed wide variation due to differences in study populations and histologic distributions. Comparative details are presented in Supplementary Table S1 and illustrated in Figure 1.

This narrative review aims to synthesize the current evidence on the prevalence, diagnosis, management strategies, and future directions for both primary and secondary cardiac tumors. The review is organized into sections on diagnostic approaches (clinical, imaging, and biopsy), management of benign and malignant tumors, prognosis, and future innovations in the field.

## 2. Diagnosis

The diagnosis of cardiac tumors relies on a combination of clinical evaluation, imaging techniques, and, when necessary, histological confirmation through biopsy.

### 2.1. Clinical Manifestations and Laboratory Correlations

The clinical presentation of cardiac tumors varies significantly between benign and malignant lesions. Benign tumors often remain asymptomatic or are discovered incidentally, while malignant tumors are more likely to present with significant symptoms such as dyspnea, chest pain, pulmonary embolism, cardiac tamponade, or systemic embolization [8].

Electrocardiographic (ECG) findings can provide additional diagnostic clues. A normal ECG is more commonly associated with benign tumors. In contrast, red flags such as tachycardia, low voltages, bradyarrhythmias, right axis deviation, or ischemic-like repolarization changes are more suggestive of malignant cardiac tumors [9].

Laboratory testing may support diagnosis in specific contexts. For example, elevated inflammatory markers and bacteremia in patients with prosthetic valves suggest infective endocarditis, while certain cardiac tumors in children, such as rhabdomyomas, may be associated with genetic syndromes like tuberous sclerosis complex (manifested by ash-leaf spots, facial angiofibromas, and cortical tubers) [10].

In patients with known primary cancers and widespread metastatic disease, a cardiac mass is often presumed to be metastatic, especially when consistent with systemic disease burden [3,11,12].

### 2.2. Non-Invasive Imaging Assessment

Transthoracic echocardiography (TTE) is typically the first-line imaging modality due to its wide availability, cost-effectiveness, and utility in hemodynamic assessment [8]. Transesophageal echocardiography (TEE) provides superior resolution, especially for characterizing valvular lesions and left atrial tumors [8]. In addition to anatomic localization, echocardiography may offer valuable clues regarding the likelihood of malignancy. Certain echocardiographic parameters—such as mass size, mobility, infiltration of surrounding structures, and irregular margins—can serve as markers of malignancy [12].

One recently validated tool, the Diagnostic Echocardiographic Mass (DEM) score, combines several of these echocardiographic features into a practical scoring algorithm to help differentiate benign from malignant cardiac tumors. A DEM score > 6 points has demonstrated strong diagnostic performance in suggesting malignancy and can help guide further imaging and treatment strategies [12,13]. The individual components and scoring criteria of the DEM score are summarized in Supplementary Table S2.

Cardiac magnetic resonance imaging (CMR) is considered the gold standard for tissue characterization of cardiac masses. CMR offers multiparametric assessment, including T1 and T2 signal characteristics, perfusion, and late gadolinium enhancement, allowing distinction between benign and malignant lesions with high diagnostic accuracy. Paolisso et al. reported near-perfect performance (AUC 0.976) using a multiparametric CMR approach [8,12,13].

Computed tomography (CT) is a valuable alternative for patients unable to undergo MRI and provides excellent spatial resolution, especially for evaluating the extent of mass infiltration and the presence of calcification [14]. 18F-fluorodeoxyglucose positron emission tomography (18F-FDG PET) is particularly useful for detecting metabolically active lesions and for staging, especially in cases of suspected metastases [10,15].

A multimodal imaging strategy, tailored to clinical presentation, patient-specific contraindications, and institutional resources, is often essential for accurate diagnosis [10,15,16]. Recent expert guidelines advocate for an integrated approach combining echocardiography, CMR, CT, and PET to optimize diagnostic confidence and clinical decision-making [16,17,18,19] (Table 1).

### 2.3. Role of Biopsy in Cardiac Tumors

While non-invasive imaging modalities such as echocardiography, cardiac MRI, and CT often provide sufficient information for diagnosis and treatment planning, biopsy remains the definitive method for histologic confirmation in cases where malignancy is suspected or when systemic therapy depends on accurate tumor classification. The decision to pursue biopsy must balance the potential diagnostic value against the procedural risks, particularly in anatomically complex or high-risk cardiac locations [22].

Biopsy may be omitted when there is a high clinical suspicion of non-neoplastic etiologies such as thrombus or infective endocarditis, especially when the modified Duke criteria are fulfilled [23]. Similarly, benign tumors like myxomas can often be diagnosed confidently based on imaging and clinical features alone. Conversely, when malignancy is suspected, tissue diagnosis becomes essential to guide oncologic planning, including the use of neoadjuvant chemotherapy or the decision to avoid unnecessary surgery in cases such as cardiac lymphoma.

When biopsy is indicated, the choice of technique depends on tumor location and patient factors. For right-sided intracavitary masses, endomyocardial biopsy is commonly performed via a percutaneous transvenous approach through the internal jugular or femoral vein [24]. Left-sided or extracavitary lesions that are inaccessible via percutaneous routes may require a surgical biopsy, typically performed through a mini-thoracotomy or median sternotomy, and often under cardiopulmonary bypass to ensure adequate exposure and safety [25,26]. In many cases, especially when resection is already planned, biopsy is performed intraoperatively at the time of tumor excision, either as a frozen section to guide surgical margins or for definitive histopathology [27].

**Figure 2:**
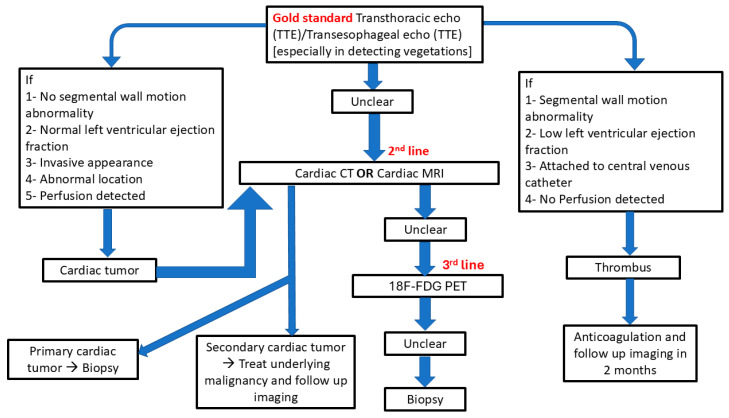
Guidelines for diagnosis of cardiac masses.

The risks of cardiac biopsy include bleeding, cardiac tamponade, arrhythmias, and embolization, with higher risk associated with tumors located near coronary vessels, the left atrium, or other structurally sensitive areas. Multidisciplinary evaluation is essential to determine whether biopsy is warranted and, if so, to select the safest and most effective approach for obtaining diagnostic tissue [22]. Guidelines for diagnosis of cardiac masses and different characteristics are shown in Figure 2, Table 2, and Supplementary Figures S1–S3.

## 3. Management of Benign Cardiac Tumors

Surgical excision remains the cornerstone of treatment for most benign cardiac tumors. Complete resection is usually curative and associated with excellent long-term outcomes [4,28]. Serial echocardiographic follow-up is recommended postoperatively to monitor for recurrence [4,28].

### 3.1. Surgical Techniques

The approach to tumor excision depends largely on the tumor location. Left atrial tumors are typically excised through a left atriotomy or superior transseptal approach, whereas right atrial tumors are accessed via right atriotomy [29]. In cases involving cardiac valves, primary repair or replacement may be necessary.

### 3.2. Safety Margins

Achieving clear surgical margins (R0 resection) is important to reduce recurrence risk, even in benign tumors like myxomas [28]. While the ideal margin remains debated, a 2–5 mm tissue buffer is commonly targeted [30,31].

Li et al. found that R0 resection significantly improved survival (58 vs. 11 months) and recurrence-free interval (36 vs. 6 months) compared to R1 (*p* < 0.001), with no survival difference between R1 and R2. They recommended aggressive attempts to achieve R0 and multimodal therapy after R1/R2 resections [32]. In contrast, Chan et al. observed no survival difference across R0–R2 resections in 122 patients, possibly due to universal chemotherapy use regardless of margin status [33].

### 3.3. Special Considerations in Pediatric Populations

In children, certain benign tumors, such as rhabdomyomas, may regress spontaneously and may not require surgical intervention unless they cause significant symptoms [22]. Close imaging surveillance is typically sufficient in asymptomatic pediatric patients.

### 3.4. Outcomes

Postoperative complications are infrequent; arrhythmias are the most common, typically managed medically [29]. Long-term survival rates following benign tumor resection are excellent, approximating survival rates of the general population in matched cohorts [30].

## 4. Management of Malignant Cardiac Tumors

Malignant cardiac tumors, primarily sarcomas and lymphomas, require aggressive multimodal therapy due to their poor prognosis [11,32,34].

### 4.1. Resectable Malignant Tumors

Whenever possible, complete surgical resection (R0) remains the primary goal, significantly improving survival compared to incomplete resections (R1 or R2) [32].

Complex resections may involve heart autotransplantation, an advanced surgical technique in which the heart is explanted, the tumor resected ex vivo, and the heart subsequently reimplanted [35]. This approach is particularly beneficial for tumors located in anatomically difficult areas such as the left atrium, mitral valve, or pulmonary veins, where in situ resection would risk incomplete excision or structural damage.

**Figure 3:**
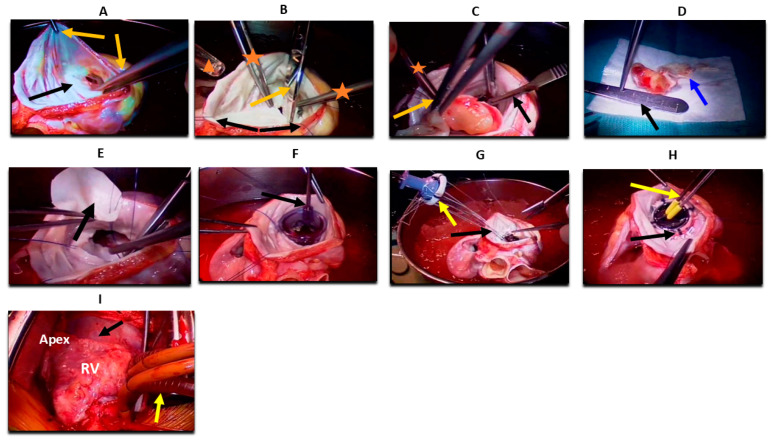
Heart autotransplantation for primary cardiac malignancy (**A**) Heart ex vivo placed in ice; the left atrium is opened (black arrow). Two forceps (orange arrows) are retracting the left atrium for proper exposure, preparing for stay sutures to maximize exposure and facilitate stability of the heart. (**B**) Stay sutures are seen peripherally (black arrows). Scissors are used to excise the anterior mitral leaflet (orange arrow). Two forceps of the first assistant are visible inside the field (orange asterisk), and the head of a sucker is seen on the side of the image (triangle). (**C**) A number 15 blade is used to excise the tumor from the papillary muscle (black arrow). One forceps is holding the tumor (orange arrow), and the other is retracting the atrial wall (asterisk). (**D**) The tumor is completely excised and placed on a white sterile dressing, measuring ~4 cm in size (black arrow). Intimate attachment to the anterior mitral leaflet is visible (blue arrow). (**E**) Forceps holding a pericardial patch (black arrow) and sewing it with running Prolene sutures in a circumferential fashion into a left ventricular defect left after complete tumor resection. (**F**) A mitral valve sizer is shown in the annulus to assess the exact size of the needed valve (black arrow). (**G**) A prosthetic valve is held with Ethibond sutures (yellow arrow) sewn through and to the annulus (black arrow). (**H**) The mitral valve prosthesis is shown in place (black arrow), with a valve tester (yellow arrow). (**I**) The heart is reimplanted into the pericardial space (black arrow). The left atrium, vena cavae, aorta, pulmonary artery, and bypass cannulas are reanastomosed. Bicaval venous cannulas are visible (yellow arrow). RV: right ventricle.

Studies have shown promising outcomes for autotransplantation, particularly when pneumonectomy is not required. However, combined autotransplantation and pneumonectomy carry high surgical mortality and are now considered contraindicated by some authors. In such cases, a two-stage approach may be employed to reduce perioperative risk [33]. Compared to orthotopic heart transplantation, autotransplantation avoids the need for immunosuppression, making it a favorable option for patients with aggressive histologies like angiosarcoma [32]. Detailed surgical steps, outcomes, and case series are summarized in Figure 3, Supplementary Tables S3 and S4.

Neoadjuvant chemotherapy or radiotherapy may improve resectability and survival in selected patients by downstaging tumors before surgery [36,37]. Following incomplete resection (R1 or R2), multimodal adjuvant therapy is recommended to address residual disease [32].

### 4.2. Non-Resectable Malignant Tumors

Tumors deemed non-resectable due to extensive local invasion, metastasis, or poor patient condition require alternative strategies:

- **Palliative Chemotherapy and Radiotherapy**: Systemic therapy aims to prolong survival and control symptoms [11,38].
- **Heart Transplantation**: Considered in select cases without metastasis; however, outcomes remain controversial due to high recurrence and the effects of immunosuppression [37,38,39,40,41].
- **Heart Autotransplantation:** It may also be considered in borderline resectable cases where standard approaches are not feasible, and there is no evidence of systemic spread. (See Section 4.1 and Supplementary Table S4 for detailed criteria and outcomes) [35].

Decision-making must be multidisciplinary, weighing surgical feasibility, systemic disease burden, and patient functional status.

### 4.3. Management of Oligometastatic Cardiac Tumors

Oligometastatic disease represents an intermediate state between localized and widely metastatic cancer, typically defined by the presence of five or fewer metastatic lesions. Although rare, oligometastatic cardiac tumors may offer opportunities for aggressive, potentially life-prolonging treatment [10]. A multimodal approach involving surgical resection, stereotactic body radiotherapy (SBRT), and systemic therapies—such as chemotherapy, targeted agents, or immunotherapy—is often warranted depending on tumor location, burden, and patient fitness [38,42,43,44,45,46,47].

Prognostic factors that favor aggressive management include a well-controlled primary tumor, low nodal stage (N0 or N1), and a disease-free interval (DFI) greater than 6 to 12 months [48]. A longer DFI is generally associated with more indolent tumor biology and improved survival outcomes following local treatment. These parameters may help guide patient selection for surgery or SBRT [34,48].

Individual case reports of oligometastatic cardiac tumors, treatment strategies, and outcomes are summarized in Supplementary Table S5.

### 4.4. Management of Cardiac Metastases

Cardiac metastases are significantly more common than primary cardiac tumors and are often detected incidentally or during advanced cancer staging. The most frequent primary tumors associated with cardiac metastases include lung carcinoma (particularly adenocarcinoma and small cell lung cancer), breast carcinoma, hematologic malignancies (especially lymphomas and leukemias), melanoma, renal cell carcinoma, and esophageal cancer [10,16].

These malignancies may reach the heart through one of three main routes: direct extension (e.g., lung or breast tumors infiltrating the pericardium), lymphatic spread (e.g., from breast or mediastinal cancers into the pericardium), and hematogenous dissemination (common with melanoma, renal cell carcinoma, and sarcomas) [6,7,16]. The pericardium is the most commonly involved site, followed by the myocardium and endocardium.

Clinical manifestations of cardiac metastases vary by location and burden of disease but may include pericardial effusion with tamponade, arrhythmias, obstruction of inflow or outflow tracts, or nonspecific symptoms such as fatigue or dyspnea. Diagnosis typically relies on multimodal imaging, including echocardiography, cardiac MRI, and 18F-FDG PET-CT, the latter of which is particularly helpful in detecting metabolically active metastatic lesions [10,15].

Management is generally palliative and tailored to symptom control. Pericardiocentesis or surgical pericardial window is indicated in cases of tamponade, and intrapericardial administration of chemotherapeutic agents or radioisotopes may help prevent recurrence [49]. For patients with life-threatening arrhythmias due to metastatic infiltration, radiofrequency ablation may offer symptomatic relief [50,51]. Antiarrhythmic medications are often required for rhythm stabilization. Rarely, surgical debulking may be considered for isolated, symptomatic cardiac metastases in otherwise stable patients [50,52,53]. In select cases of limited metastasis (oligometastatic disease), a more aggressive approach combining local and systemic therapies may be justified (see Section 4.3).

## 5. Prognosis

Prognosis in cardiac tumors depends heavily on tumor type, resectability, and completeness of surgical excision. Benign tumors generally have excellent outcomes, while malignant tumors—particularly sarcomas—are associated with poor survival despite aggressive treatment.

### 5.1. Benign Tumors

Most benign tumors, such as myxomas and lipomas, have favorable long-term outcomes following complete surgical resection (R0). Recurrence is rare with adequate excision, and postoperative survival approximates age-matched general population rates [30,54,55,56]. Pediatric benign tumors such as rhabdomyomas often regress spontaneously, further contributing to an excellent prognosis in this subgroup [22].

### 5.2. Malignant Tumors

Primary malignant cardiac tumors, such as angiosarcomas and undifferentiated sarcomas, carry a dismal prognosis. Median survival typically ranges from 6 to 12 months for patients with unresected or incompletely resected tumors. However, complete surgical resection (R0) has been associated with significantly improved outcomes. In a study by Li et al., patients who underwent R0 resections had a median survival of 58 months compared to only 11 months for those with R1 or R2 resections (*p* < 0.001) [32]. Conversely, Chan et al. reported no significant difference in survival between R0, R1, and R2 resections in a cohort where all patients received chemotherapy, suggesting that systemic therapy may play a crucial role in mitigating the negative impact of incomplete resection [33].

### 5.3. Prognosis in Oligometastatic Disease

In selected patients with oligometastatic cardiac involvement, prognosis is more favorable when the primary tumor is well controlled, nodal disease is limited (N0 or N1), and the disease-free interval (DFI) exceeds 6–12 months [48]. A longer DFI often reflects less aggressive tumor biology and a better response to localized therapies such as surgical resection or stereotactic body radiotherapy (SBRT). For instance, patients with brain metastases and a DFI greater than 360 days, or with adrenal metastases and a DFI exceeding 6 months, demonstrated improved survival outcomes [48]. In one reported case of cardiac metastasis from lung adenocarcinoma, treatment with SBRT resulted in a complete metabolic response on PET imaging and sustained tumor control lasting over 18 months [38].

### 5.4. Metastatic Cardiac Tumors

Cardiac metastases generally indicate advanced-stage malignancy with a poor prognosis. Survival is typically determined by the behavior of the primary tumor rather than the cardiac involvement itself. Palliative care remains the mainstay of treatment in such cases, and survival is often measured in weeks to months [2,50].

## 6. Future Directions

Advances in cardiac tumor management increasingly depend on the integration of artificial intelligence (AI), precision diagnostics, and collaborative global data sharing. AI-enhanced imaging, including machine learning algorithms applied to echocardiography and cardiac MRI, holds significant promise in improving diagnostic accuracy and enabling earlier tumor detection. One such tool is the echocardiographic DEM score, a multiparametric algorithm that can help differentiate benign from malignant cardiac tumors and guide further diagnostic steps [12].

Genomic profiling and molecular diagnostics are expected to refine treatment planning, particularly for aggressive histologies such as sarcomas. As more molecular targets are identified, the use of precision medicine—including targeted therapies and immunotherapies—may help individualize treatment regimens and improve survival.

Innovations in surgical techniques, such as 3D modeling and minimally invasive tumor resections, could enhance operative precision and reduce complications. Refinement of criteria for advanced interventions like heart autotransplantation will also be critical for selecting suitable candidates and improving outcomes.

Furthermore, international registries and prospective databases are urgently needed to improve the understanding of these rare tumors. Pediatric-focused studies should address the unique tumor biology and clinical course of cardiac tumors in children, especially those associated with syndromic conditions like tuberous sclerosis.

Addressing global disparities in access to diagnostic imaging and specialized care remains a key priority. Equitable distribution of resources such as cardiac MRI and PET-CT, particularly in low- and middle-income settings, will be essential to improve diagnostic capabilities and reduce outcome gaps.

Finally, future clinical trials and treatment strategies must prioritize patient-centered outcomes, including quality of life, functional recovery, and long-term surveillance. Public health efforts should promote awareness of early cardiac tumor symptoms—such as unexplained arrhythmias or embolic events—to support earlier diagnosis and referral to specialized centers.

## 7. Conclusions

Cardiac tumors, though rare, present substantial diagnostic and therapeutic challenges due to their diverse pathology and the critical structures they involve. Differentiating benign from malignant lesions is essential, as management strategies and prognostic implications vary significantly. While echocardiography remains the first-line diagnostic tool, advanced imaging modalities such as cardiac MRI and PET-CT have greatly enhanced diagnostic accuracy and risk stratification.

For resectable tumors, particularly benign lesions like myxomas, surgical excision is usually curative with excellent long-term outcomes. Malignant tumors, however, often require a multimodal approach involving surgery, chemotherapy, radiotherapy, or innovative techniques like heart autotransplantation. Prognosis in malignant cases remains guarded, although survival improves with complete resection and carefully selected aggressive interventions in oligometastatic settings.

Progress in cardiac oncology will depend on standardizing treatment algorithms, integrating novel technologies such as artificial intelligence and genomic profiling, and expanding global registries to better understand rare tumor types. Special emphasis is needed on pediatric populations and on bridging diagnostic and therapeutic disparities across regions.

Ultimately, a multidisciplinary, patient-centered approach—supported by technological innovation and collaborative research—is essential to improve outcomes and reduce recurrence in patients with primary or metastatic cardiac tumors.

## Figures and Tables

**Table 1:** Different characteristics of some cardiac tumors [3,20,21].

Tumor	Histopathology	Need for Biopsy	Surgery	ECHO	Cardiac MRI (CMR)	CT Findings	18F-FDG PET Findings
**Myxoma**	- Spindle or stellate cells - Pseudo-vascular structure - Hemorrhage - Dystrophic calcification	Not needed	Complete resection	- Narrow stalk - Fossa ovalis - Isoechogenic	- Iso- to hypointense on T1, hyperintense on T2 - Heterogenous enhancement with contrast	Well-defined, hypodense mass; may show calcification	Mild to moderate uptake; not typically FDG-avid
**Lipoma**	- Mature adipocytes	Not needed	For severely symptomatic cases	- Intracavitary (homogenous and hypoechoic) - Pericardial (hypoechoic)	- Hyperintense bright signal in T1 and T2, reduced with fat suppression technique - No enhancement	Homogeneous fat density (−80 to −120 HU)	No uptake (photopenic)
**Fibroma**	- Fibroblasts and collagen - Elastic fibers	Not needed	For severely symptomatic cases	- Homogenous, brighter than surrounding myocardium	- Hypointense in all T1, T2 - Late contrast enhancement	Well-defined, hyperdense; possible calcification	Generally low uptake
**Rhabdomyoma**	- Spider cells (vacuolated enlarged cardiac myocyte with clear cytoplasm due to abundant collagen)	Not needed	- Spontaneous regression - For severely symptomatic cases	- Bright echogenic ventricular mass, embedded in wall or protruding into cavity	- Isointense in T1 - Iso to hyperintense T2 - No enhancement	Soft-tissue density within myocardium; non-calcified masses	Typically, low or absent uptake
**Angiosarcoma**	- High vascularity - Myocardial infiltration - Pleomorphic, mitosis, and necrosis	If possible	- Complete excision - Debulking to improve symptoms	- Dense, irregular mass, non-mobile, broad-based, with endocardial to myocardial extension	- Heterogenous in T1 and T2 - Heterogenous contrast enhancement (sunray appearance)	Large, lobulated, heterogeneous mass with irregular borders; may invade pericardium	High uptake; intense FDG avidity
**Leiomyosarcoma**	- Spindle cells with blunt nuclei - Necrosis, mitosis - Epithelioid regions	If possible	- Complete excision - Debulking to improve symptoms	- Echogenic mass of the cardiac chambers	- **T1:** isointense - **T2:** hyperintense - Heterogeneous early and late enhancement	Infiltrative soft-tissue mass; may extend into pulmonary veins	High FDG uptake
**Rhabdomyosarcoma**	- Embryonal type with rhabdomyoblasts - Abundant glycogen, desmin, myoglobin, myogenin	If possible	- Complete excision - Debulking to improve symptoms	- Intracardiac mass	- Variable signal intensity	Poorly defined, enhancing soft-tissue mass	High FDG uptake
**Undifferentiated sarcoma**	- Plump spindle cells with frequent mitotic activity - Pleomorphic nuclei	If possible	- Complete excision - Debulking to improve symptoms	- Broad-based mass with heterogenous echogenicity	- Isointense on T1 and hyperintense on T2 - Heterogeneous, delayed enhancement	Irregular soft-tissue mass; may involve pulmonary veins or atrial wall	High FDG uptake
**Primary cardiac lymphoma**	- Large B cell lymphoma (most common) - Burkitt lymphoma - Low-grade B cell - T cell lymphoma	Required	No role for surgery	- Homogeneous, infiltrating mass leading to ‘wall thickening’ and restrictive hemodynamics - Nodular masses intruding into heart chambers	- Atrial, particularly right atrial predominant location. - Variable signal intensity and contrast enhancement	Soft-tissue density; right atrial or pericardial mass common	Markedly FDG-avid; useful for diagnosis and response assessment
**Metastasis**	Dependent on primary tumor	Required at site of primary tumor	- Debulking to improve symptoms	- Infiltrating malignant cells - Necrosis	- Multiple lesions - Pericardial effusions	Nodular or infiltrative lesions, often associated with lung or breast cancer	High FDG uptake in most cases, depending on primary tumor type

**Table 2:** Resection and Reconstruction in cardiac tumors.

Zone		Affected Organ	Site	Resection Extent	Reconstruction
1	Great vessels	- Pulmonary artery (more common) - Aorta	- Usually begins at the level of the valve and extends distally	- resection of all visible tumor bulk - resection of ventriculo-aortic/pulmonary valve - Great vessel resection - Frozen section is carried out to help in the extent of resections	- Distal ventricular outflow tract - Cardiac valve - Great vessel - Hilum in case of pulmonary artery sarcoma - Reconstruction using Dacron, homograft, or pericardium is well tolerated
2	Venous Disease	- SVC	- Pulmonary tumors with SVC involvement	- Resected en-bloc. - Intraoperative tumor-free margins by frozen section.	- With ringed PTFE (standard). - Pericardial tubes
		- IVC	- Primary IVC tumor - IVC involvement with renal cell carcinoma (the most common reason for cardiac surgical intervention on the IVC)	- IVC resection using total circulatory arrest - Palliative resection (in IVC sarcomas)	- By synthetic tubular material
		Pulmonary vein	- Pulmonary tumors - Mediastinal tumors	- Resection on cardiopulmonary bypass after neoadjuvant therapy to allow adequate atrial margins	
3	Atrial tumors	Left atrium	- Interatrial septum - Both sides of the septum - Anywhere in the atrium - Atrial side of the valves	- Resection of the whole tumor with a “donut” of tissue surrounding the primary lesion to ensure adequate resection	- By preserved bovine pericardium - Orthotopic heart transplantation - Autotransplantation
4	Ventricular tumors	Left ventricle		- Resection only in case there is a stalk - Resection is very difficult	- Heart transplantation
5	Cardiac valves	Any valve papillary fibroelastomas (aortic followed by mitral valve)		- Resection	- Primary repair or replacement of the valve

IVC: inferior vena cava; PTFE: polytetra-fluro-ethylene; SVC: superior vena cava.

## Supplementary Materials

The following supporting information can be downloaded at: https://www.mdpi.com/article/10.3390/jcm14103392/s1. Ref. [57] is cited in the Supplementary Table S1.
