# Supplementary material for: Prevalence, Diagnosis, and Treatment of Cardiac Tumors: A Narrative Review
Source: J Clin Med. 2025 May 13;14(10):3392. doi: 10.3390/jcm14103392 (PMC12111963; doi:10.3390/jcm14103392)
Supplement: Supplementary file 1 [file jcm-14-03392-s001.zip › jcm-3616328-supplementary.pdf]

Journal of
Clinical Medicine

MDPI

Review

Prevalence, diagnosis, and treatment of cardiac tumors; a narrative review

Mohamed Rahouma MD, PhD [1,2], Hosny Mohsen MD [3], Mahmoud Morsi MD [1], Sherif Khairallah MD [1,2], Lilian Azab [1], Maya Abdelhemid [1,4], Akshay Kumar MD [5], Magdy M El-Sayed Ahmed MD and MS [6,7]

1- Cardiothoracic Surgery Department, Weill Cornell Medicine/New York Presbyterian Hospital, New York, USA
2- Surgical Oncology Department, National Cancer Institute, Cairo University, Egypt Cardiothoracic Surgery
3-Department of Cardiothoracic Surgery, Faculty of Medicine, Beni Suef University, Beni Suef 2721562, Egypt
4-Department of Biology and Psychology, Stony Brook University, Stony Brook, NY, USA
5- Cardiothoracic Surgery Department, NYU Langone Health, New York, NY, United States.
6- Cardiothoracic Surgery Department, Mayo Clinic, Jacksonville, FL 32224, USA.
7-Surgery Department, Faculty of Medicine, Zagazig University, Zagazig 44519, Egypt.

* Correspondence: **authors:** Mohamed Rahouma MD, PhD, Department of Cardiothoracic Surgery, Weill Cornell Medicine, 525 E 68th St, New York, NY 10065, Telephone: +1 212 746 9440 Fax: +1 212 746 8080, E-mail: mhmdrahouma@gmail.com ; mmr2011@med.cornell.edu

Journal of Clinical Medicine MDPI

Supplementary materials

Supplementary Table S1: Prior meta-analyses-reported outcomes

Outcomes	Rahouma et al 2020	He et al 2017	Silva et al 2015	Tzani et al 2017 (pediatric)
**Prevalence of PMCT among PCT**	10.83% [9.11; 12.83]	9.9% [8.4; 11.4]	-----	-----
**Prevalence of PMCT among CT**	10.16% [8.54; 12.06]	-----	-----	-----
**Prevalence of SMCT among CT**	9.91% [7.33; 13.26]	-----	-----	-----
**Prevalence of myxoma among PCT**	61.93% [55.74; 67.76]	-----	-----	-----
**Prevalence of myxoma among CT**	58.14% [51.95; 64.09]	-----	-----	-----
**Incidence of short term (1 month) mortality in CT**	5.90% [4.70; 7.39]	-----	-----	6.7%
**Incidence rate of late mortality in all CT**	2.55% [1.76; 3.72]	-----	-----	6.4%
**Incidence rate of late mortality in Benign CT**	0.79% [0.46; 1.37]	-----	-----	8.2%
**Incidence rate of late mortality in PMCT**	14.77% [9.32; 23.40]	-----	-----	66.3%
**Prevalence of HTX for PMCT among total PMCT**	13.85% [6.51; 27.05]	-----	-----	-----
**Prevalence of HTX for PMCT among total CT**	1.44% [0.67; 3.08]	-----	-----	-----
**Prevalence of HTX for PMCT among total MCT(1ry,2ry)**	8.98% [4.21; 18.14]	-----	-----	-----
**Prevalence of HTX among total CT**	2.45% [1.36; 4.38]	-----	-----	-----
**Prevalence of CAD in myxoma patients**	-----	-----	20.7% [12.0; 32.0]	-----

1ry: primary, 2ry: Secondary, CAD: coronary artery disease, CT: cardiac tumors, HTX: heart transplantation, MCT: malignant cardiac tumors, MTA: meta-analysis, PCT: primary cardiac tumors. PMCT: primary malignant cardiac tumors, SMCT: secondary malignant cardiac tumors,

Journal of Clinical Medicine

MDPI

Supplementary table S2: The Diagnostic Echocardiographic Mass (DEM) score details (12).

The **Diagnostic Echocardiographic Mass (DEM) score** is a validated multiparametric tool designed to differentiate between benign and malignant cardiac masses using transthoracic echocardiography (TTE). Developed through a comprehensive study at the University of Bologna, the DEM score assists clinicians in risk stratification and decision-making regarding further diagnostic evaluations.

Components of the DEM Score:

The DEM score incorporates six echocardiographic parameters, each assigned a specific point value based on their association with malignancy:

- **Infiltration into adjacent structures**: 2 points
- **Polylobate (multi-lobed) shape**: 2 points
- **Moderate to severe pericardial effusion**: 2 points
- **Inhomogeneous echotexture**: 1 point
- **Sessile implantation (broad-based attachment)**: 1 point
- **Non-left cardiac localization**: 1 point

The total score ranges from 0 to 9, with higher scores indicating a greater likelihood of malignancy.

Clinical Application:

- **Scores ≥5**: High probability of malignancy
- **Scores 3–4**: Intermediate risk; further imaging (e.g., cardiac MRI or PET/CT) is recommended
- **Scores <3**: Suggestive of benign pathology

In the validation cohort, a DEM score of ≥3 demonstrated a diagnostic accuracy exceeding 90% for identifying malignant cardiac masses. Additionally, higher DEM scores correlated with poorer survival outcomes, underscoring the score's prognostic value.

Journal of
Clinical Medicine

MDPI

Supplementary Table S3: Operative Steps of Heart Autotransplantation

Referenced in Section 4.1 of the manuscript.

Heart autotransplantation is a technically complex surgical approach used for resection of centrally located or infiltrative cardiac tumors that are not amenable to conventional in situ resection. The following steps outline the operative procedure based on the technique described by Ramlawi et al. (31):

1. **Surgical Access**
   A median sternotomy is performed to expose the heart and great vessels.
2. **Vessel Mobilization and Cannulation**
   The superior and inferior vena cava (IVC) are extensively mobilized. Venous cannulation is performed via bicaval cannulation (high in the SVC and low in the IVC). Arterial cannulation is typically performed via the distal ascending aorta, though femoral cannulation may be used in repeat operations.
3. **Initiation of Cardiopulmonary Bypass (CPB)**
   CPB is initiated, and the ascending aorta and pulmonary artery are mobilized.
4. **Cardiectomy**
   After administration of antegrade cardioplegia, the heart is explanted via transection of the SVC, IVC, aorta, and pulmonary artery. The left atrium is divided just anterior to the pulmonary veins.
5. **Ex Vivo Tumor Resection**
   With the heart placed in an ice bath, tumor resection is performed ex vivo under direct visualization. Margins are assessed, and myocardial defects are repaired using bovine pericardium.
6. **Valve Assessment and Replacement**
   All valves are inspected. If required, prosthetic valve replacement is performed using standard techniques.
7. **Repeat Cardioplegia and Hemostasis**
   Repeat cardioplegia doses are administered directly into the coronary ostia. Hemostasis is secured via direct suturing of bleeding sites.
8. **Reimplantation of the Heart**
   The heart is reimplanted into the pericardial cavity. Anastomoses are performed in a bicaval fashion to preserve coronary sinus drainage, differing from standard orthotopic transplantation.
9. **Special Considerations**
   Reconstruction of the IVC is often the most technically challenging step due to limited residual tissue. A Gore-Tex graft (W.L. Gore & Associates) may be required if tension-free reanastomosis is not possible.

Journal of
Clinical Medicine

MDPI

Supplementary Table S4: Summary of Heart Autotransplantation for Malignant Cardiac Tumors: Indications, and Outcomes

Study / Author	Patient Population	Indication / Tumor Site	Key Findings	Notes / Limitations
**Ramlawi et al. (31)**	34 patients (27 malignant, 7 benign)	Mostly LA / MV / PV	OS for malignant tumors: 81% (30d), 46% (1yr), 28% (2yr). Pneumonectomy contraindicated (43% 30d mortality).	Largest reported series; recommends adjuvant chemo
**Edward et al. (29).**	6 patients	Requiring both cardiac tumor resection and pneumonectomy	Two-stage approach reduced mortality to 0%	Avoids complications from CPB-induced coagulopathy
**Li et al. (26).**	Meta-analysis context	Angiosarcoma	Autotransplantation may be superior to transplantation by avoiding immunosuppression	Tumor-limited LA candidates may benefit

LA: left atrium; MV: mitral vein; PV: pulmonary vein

Journal of Clinical Medicine

MDPI

Supplementary Table S5. Reported Cases of Oligometastatic Cardiac Tumors and Their Management

Reference	Primary Tumor	Cardiac Site	Treatment Modalities	Outcome
**Linfeng et al. (43)**	Cardiac sarcoma (primary)	Right atrium and ventricle	Incomplete surgical resection (no adjuvant therapy)	Brain metastasis at 4 months; death at 10 months post-op
**Masci et al. (30).**	Hepatocellular carcinoma	Right ventricle	Surgical resection followed by chemotherapy	Local recurrence at 2 months; reduced tumor size with systemic therapy
**Tamura et al. (14).**	Hepatocellular carcinoma	Right ventricle	Radiotherapy + Lenvatinib (immunotherapy)	Tumor shrinkage and hemodynamic improvement at 3-month follow-up
**Jumeau et al. (42)**	Lung adenocarcinoma	Interventricular septum	Chemotherapy + SBRT	Complete metabolic response at 3 and 6 months; no recurrence at 18 months
**Fernandez et al. (53)**	Various primaries (retrospective)	Brain, adrenal, multiple sites	Retrospective cohort analysis of oligometastatic cases	Improved survival in patients with DFI >360 days (brain), >1 year (multiple), >6 months (adrenal)

DFI: disease free interval; SBRT: stereotactic body radiotherapy

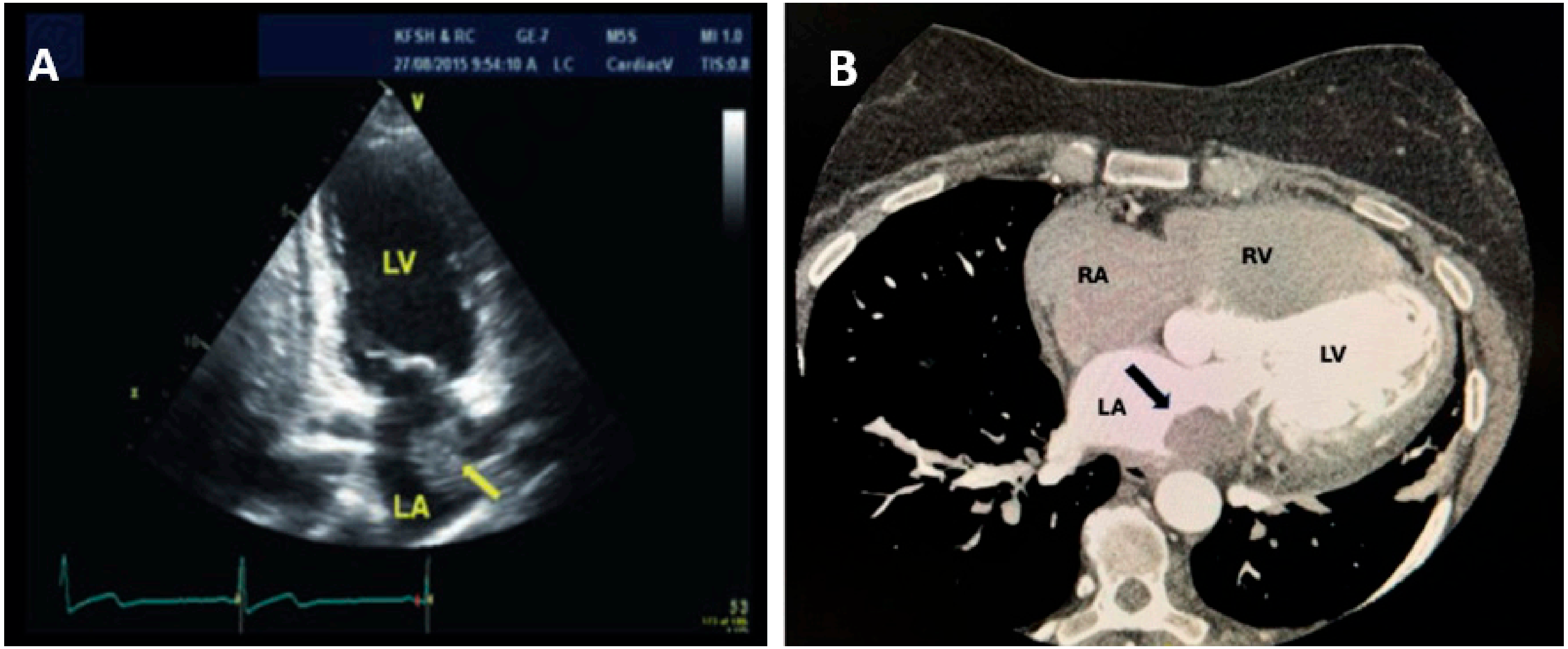

Supplementary Figure 1: a) Myxoma: Transthoracic echocardiography image showing a 2-chamber apical view with LA myxoma (Adapted from Alamro et al., *Monaldi Arch Chest Dis*, 2024;94:2768, CC BY 4.0) and B) A contrast-enhanced CT image showing an endoluminal mass in the left atrium, likely metastatic from renal carcinoma. The black arrow indicates pathological tissue within the left atrium during the arterial phase following iodinated contrast infusion. RA: right atrium; LA: left atrium; LV: left ventricle; RV: right ventricle. (Adapted from Lucà F et al., *Life*, 2023;15(2):291, licensed under CC BY 4.0.)

Journal of
Clinical Medicine

MDPI

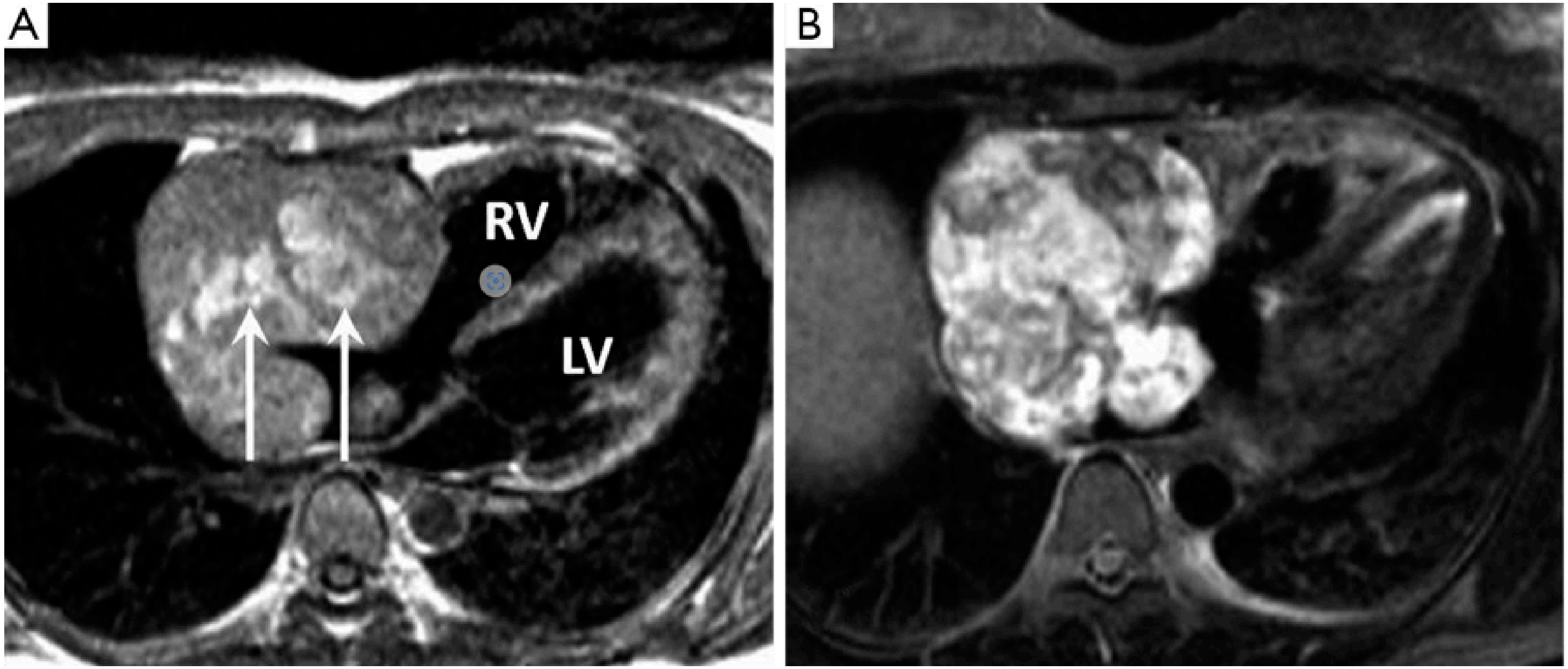

Supplementary Figure 2: Angiosarcoma. (A) Axial T1-weighted black blood image showing a large infiltrative heterogeneous signal mass. Central areas of high T1 signal suggest the presence of intra lesional haemorrhage (arrows). (B) Axial T2-weighted black blood image showing the tumour to contain areas of very high T2-signal change in keeping with oedema and some areas of low signal intensity suggestive of necrosis. RV, right ventricle; LV, left ventricle. Adapted from Hoey ETD et al., *QIMS*, 2014;4(6):489–497, used under CC BY-NC-ND 3.0.)

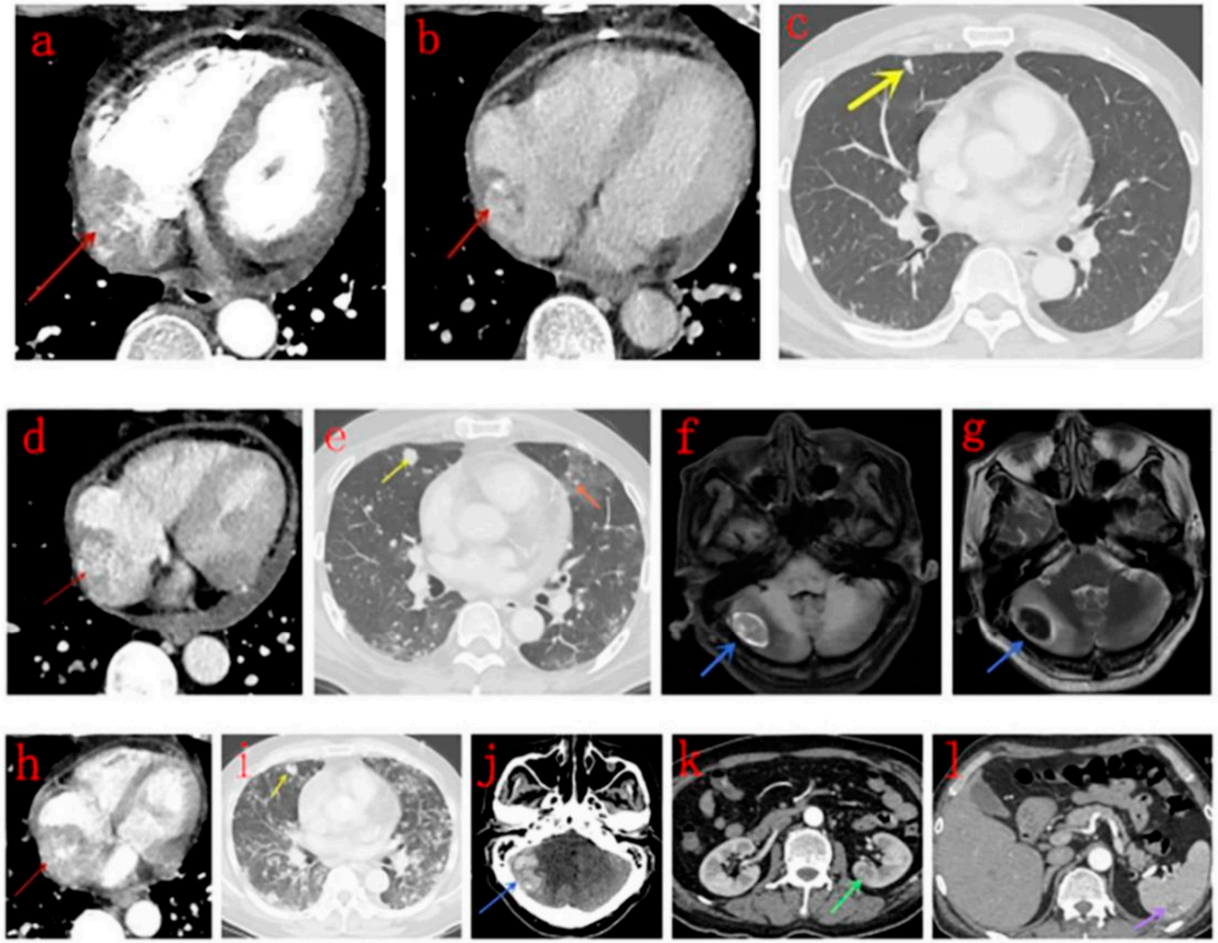

Supplementary Figure 3. Male, 52 years old, right atrium angiosarcoma. (a–c): Contrast-enhanced computed tomography (CT) scans in the arterial (a) and venous (b) phase indicates a mass with heterogeneous centripetal enhancement (red arrow), and multiple solid nodules (c) are noticed in the lungs (yellow arrow). (d–g): 1.5 months later, pericardial effusion appears (d), metastases (yellow arrow) in the lungs are more extensive, halo sign appears (e, orange arrow). Metastasis in the right side of the cerebellum (blue arrow) presents as inhomogeneous hyperintensity on T1WI (f) and hypo-intensity on T2WI (g) with

surrounding edema. (h–l): Another month later, the mass (red arrow) becomes larger, and the adjacent pericardium invasion is more extensive (h), and metastases in the lungs are much more extensive (i). The metastasis in the right side of the cerebellum (blue arrow) is larger (j). Metastases in the kidney (k, green arrow) and spleen (l, purple arrow) appear. Adapted from Chen Y et al., Diagnostics, 2020, 10(10):776, licensed under CC BY 4.0.
